# A Case of Snakebite Treated with Adrenalin Chloride

**Published:** 1903-09

**Authors:** J. D. Hooker

**Affiliations:** Alice, Texas


For Texas Medical Journal.

### A Case of Snakebite Treated with Adrenalin Chloride.

BY J. D. HOOKER, M. D., ALICE, TEXAS.

I was called to see Samuel Garcia, a Mexican boy, age 10 years, at 11 o'clock June 3rd. This boy had been struck by a rattlesnake about two hours before I saw him. Both fangs had entered anterior part of lower leg, three inches above ankle. The leg had been corded, but not sufficiently to materially restrict the circulation. Considerable swelling already existed in lower leg.

At this time his pulse was 86 per minute, soft and wavy; respiration 20; pupils slightly dilated. Patient seemed very nervous, excited and irritable. I scarified the wounds where the fangs had entered and used a cupping glass, which drew off considerable blood. I then injected 1 per cent solution pott. permang. into and around the wound.

Having just read in the Texas Medical Journal the article written by Dr. Menger, of San Antonio, in which he suggested the probable benefits to be derived from the use of adrenalin chloride in rattlesnake bites, I decided to use it in this case.

At 11 o'clock a. m., when conditions were as above given, I injected hypodermically eight minims of Parke, Davis & Co.'s 1 to 1000 solution of adrenalin chloride and one-thirtieth gr. sulph. strych.

At 3 o'clock p. m. his pulse was 104, respiration 24 per minute; his pupils still more dilated than at 11 o'clock. The leg was very tender, and swollen to knee. Had urinated twice in the four hours. I now repeated the dose of adrenalin chloride of eight minims hypodermically, with one-thirtieth gr. sulph. strych.

At 6 o'clock p. m. pulse was 90, respiration 20 per minute. Had taken a little nourishment in form of coffee and milk.

At 9 o'clock p. m. pulse 70 per minute, full and strong; respiration 18, and pupils normal. Patient had again urinated. Gave a teaspoonful of cascara evacuant. I now enveloped entire foot and leg in ointment of glyceroplasma, cotton and bandage, directed plenty of water and milk to be given, and one-thirtieth gr. sulph. strych. at 12 o'clock m. I did not repeat the adrenalin chloride at this time, as patient's condition did not indicate any need for its repetition. The further progress of this case was towards rapid recovery.

I did not know what dose of the adrenalin chloride to give and so determined to begin with small doses, watching effects, and increase dose as case seemed to demand. I would not hesitate to give much larger doses than given in this case, if symptoms seemed to demand larger.

Although I consider normal saline solution the best remedy at our command in these cases, I did not use it in this case because it was not needed.

I have restored to life one case of a child, two years old, bitten by a rattlesnake, who seemed in a moribund condition, by subcutaneous injections of normal saline solution. It should always be used in cases where symptoms are grave, and even in desperate conditions will save your patient.

I think it acts in three ways: (1) By diluting the poison in the blood, reducing its toxic effects upon the nerve centers; (2) by increasing elimination of poison by the kidneys, and (3) by its direct stimulating effects upon the heart.

I would use it by the bowels, subcutaneously, and, in extreme cases, intravenously.

The mode of death in cases of snakebite is by general vaso-motor paralysis. This being the case, adrenalin chloride suggests itself, physiologically, as the remedy to meet this condition. Next to the normal saline solution I believe it will prove our best remedy in poisonous snakebites. I, at least, expect to continue its use until satisfied as to its exact value in cases of snakebites, and I am thankful to Dr. Menger for suggesting its use in these cases.

